# Memory representations during slow change blindness

**DOI:** 10.1167/jov.24.9.8

**Published:** 2024-09-10

**Authors:** Haley G. Frey, Lua Koenig, Ned Block, Biyu J. He, Jan W. Brascamp

**Affiliations:** 1 Department of Psychology, Michigan State University, East Lansing, MI, USA; 2 Neuroscience Institute, New York University Grossman School of Medicine, New York, NY, USA; 3 Department of Philosophy, New York University, New York, NY, USA; 4 Department of Neurology, New York University Grossman School of Medicine, New York, NY, USA; 5 Department of Neuroscience & Physiology, New York University Grossman School of Medicine, New York, NY, USA; 6 Department of Radiology, New York University Grossman School of Medicine, New York, NY, USA

**Keywords:** slow change blindness, perception, memory representations

## Abstract

Classic change blindness is the phenomenon where seemingly obvious changes that coincide with visual disruptions (such as blinks or brief blanks) go unnoticed by an attentive observer. Some early work into the causes of classic change blindness suggested that any pre-change stimulus representation is overwritten by a representation of the altered post-change stimulus, preventing change detection. However, recent work revealed that, even when observers do maintain memory representations of both the pre- and post-change stimulus states, they can still miss the change, suggesting that change blindness can also arise from a failure to compare the stored representations. Here, we studied slow change blindness, a related phenomenon that occurs even in the absence of visual disruptions when the change occurs sufficiently slowly, to determine whether it could be explained by conclusions from classic change blindness. Across three different slow change blindness experiments we found that observers who consistently failed to notice the change had access to at least two memory representations of the changing display. One representation was precise but short lived: a detailed representation of the more recent stimulus states, but fragile. The other representation lasted longer but was fairly general: stable but too coarse to differentiate the various stages of the change. These findings suggest that, although multiple representations are formed, the failure to compare hypotheses might not explain slow change blindness; even if a comparison were made, the representations would be too sparse (longer term stores) or too fragile (short-lived stores) for such comparison to inform about the change.

## Introduction

Change blindness is a robust phenomenon characterized by observers’ failure to notice seemingly obvious changes in their visual input. In most cases of change blindness in the literature, such unnoticed changes coincide with other visual transients. For example, observers may miss the disappearance or replacement of a scene element that co-occurs with the simultaneous appearance of small markings elsewhere in the scene (so-called “mud-splashes”) (O'Regan, Rensink, & Clark, 1999) or with a brief blanking of the whole scene, either due to a blank frame inserted between frames (interstimulus interval) (Rensink, O'Regan, & Clark, 1997) or due to a blink or eye movement (Grimes, 1996; McConkie & Currie, 1996; O'Regan, Deubel, Clark, & Rensink, 2000).

The change blindness phenomenon raises questions about the brain's representation of current and prior visual information. A comparison between representations of the pre-change stimulus state and the post-change stimulus state should readily alert observers to the change, even if they miss seeing the change occur; however, the nature of such representations is heavily debated. Early interpretations of change blindness centered on the idea that only a small fraction of the visual world is stored beyond the present moment (O'Regan, 1992), and the resulting lack of stored information would explain why even substantial changes may go unnoticed. The disruption of attentional mechanisms during classic change blindness suggests that a lack of attention to pre-change information can explain the failure to notice changes (Awh, Vogel, & Oh, 2006; Caplovitz, Fendrich, & Hughes, 2008; Chun & Turk-Browne, 2007). Several other interpretations do allow for the initial formation of a memory representation of the pre-change scene and propose that change blindness occurs when this representation fades or is overwritten by a representation of the new, post-change scene (Beck & Levin, 2003; Irwin, 1992; Noë, Pessoa, & Thompson, 2000; O'Regan & Noë, 2001; Rensink et al., 1997). More recent work has provided evidence that, in fact, both pre-change and post-change representations can exist simultaneously even when the observer does not notice the change (Beck & Levin, 2003; Hollingworth & Henderson, 2002; Mitroff, Simons, & Levin, 2004; Simons, Chabris, & Schnur, 2002), leading to the suggestion that the inability to notice the change may reflect a failure to compare representations rather than their absence (Mitroff et al., 2004; Smith, Lamont, & Henderson, 2012). Relatedly, there is evidence that, during change blindness, a changing item may still be registered implicitly, as indicated by an influence on subsequent behavior such as task accuracy, pupil size, or eye-movement patterns (Chetverikov, Kuvaldina, MacInnes, Jóhannesson, & Kristjánsson, 2018; Fernandez-Duque & Thornton, 2003). This is consistent with the possibility that representations of the changing item may exist, even if a change in the item remains unnoticed.

Most knowledge on change blindness and, indeed, all evidence summarized above come from studies on so-called *classic change blindness*: blindness to a sudden visual change that coincides with other visual events. But, observers have also demonstrated blindness to changes that occur without distracting events, provided that the changes unfold too slowly for a visual transient to capture attention (David, Laloyaux, Devue, & Cleeremans, 2006; Frey, Koenig, He, & Brascamp, 2024; Hollingworth & Henderson, 2004; Laloyaux, Devue, Doyen, David, & Cleeremans, 2008; Simons, Franconeri, & Reimer, 2000). In these studies, participants are presented with a stimulus in which a part of a visual scene changes, appears, or disappears slowly over the span of many seconds, and they are asked to report changes that they notice. Crucially, a large proportion of participants do not report the gradual change, thus demonstrating *slow change blindness*. These studies illustrate how robust the phenomenon is but do not attempt to quantify the visual representations involved. What is especially intriguing about such slow change blindness is that the stimuli are uninterrupted and observers have plenty of time to look for changes. As such, one would intuitively expect observers to be aware at some point that something has changed, even if they did not witness the change as it occurred. The fact that they do not underscores that our awareness may be sparser than our intuitions would suggest. Slow change blindness may also be particularly informative when it comes to furthering the understanding of visual processing in natural viewing conditions, which often involves movement of objects as well as gradual changes in, for example, viewpoint or lighting.

Very little literature on slow change blindness exists, and it is clear that some findings from work on classic change blindness cannot be directly applied here. The finding that observers maintain both a pre-change and a post-change representation side by side may not generalize to a situation where the change between the initial and final state occurs gradually via numerous intermediate steps and over a much longer period. One study shows that observers do not notice a gradual rotation in the viewpoint of a scene but do notice a reversion back to the initial view, evidence that the observer's representation of the scene is continually updated as slightly different visual information keeps reaching the senses, despite the observer's failure to notice that anything is changing (i.e., implicit updating) (Hollingworth & Henderson, 2004). Other work, however, indicates that what is represented by the end of an unnoticed slow change is not simply the most recent state of the scene but rather incorporates information from both recent and earlier steps in the changing sequence (Laloyaux et al., 2008).

To investigate what sort of representations an observer may form and retain during slow change blindness, we presented observers with images in which a large, centrally located area slowly changed color. Even though these changes are obvious when they happen quickly, when they unfold over many seconds (16 in our case) they are rarely noticed (Frey et al., 2024). These stimuli are particularly interesting for studies of slow change blindness because they elicit change blindness even when the changing scene element covers a large part of the visual field (including the center of fixation). To gain insight into the observer's representation of the changing region, we probed this representation as soon as the change had finished and the image had been removed from the screen. We did so by cueing attention to the relevant region of the scene and then showing a comparison image that could match the slowly changing region as it was at a particular timepoint during the slow change. Across the experiments that we performed, this timepoint could be the initial timepoint, the final one, or a timepoint midway in the change. For comparison, our later experiments also included conditions where the comparison image did not, in fact, match the color-changing region at any timepoint, as well as ones where the region did not actually change color. Observers reported whether the comparison image matched the cued region as it had been immediately before it disappeared. Although we asked about the final state of the region before its disappearance, the observer's comparison judgment was indicative of their internal representation of the region, which could in principle be influenced by this final state as well as by earlier stimulus states. Mindful of the fact that the mind harbors multiple memory systems, we performed three variants of the experiment, each using a slightly different cueing procedure, aimed at probing different memory systems. We hypothesized that different memory systems may be influenced by the changing stimulus information to different extents and that reports of “same” or “different” would be influenced by the memory system probed. Overall, our results are consistent with the idea that observers have access to multiple memory representations during change blindness and that some of those representations get overwritten by updated ones as the change happens. Other representations may be more stable over time, yet insufficiently detailed to alert observers that a change has happened.

## Method

### Participants

In all experiments, participants were recruited using Prolific (www.prolific.co) (Palan & Schitter, 2018), and the experiments were made available online using Pavlovia (https://pavlovia.org) (Peirce et al., 2019). Participants were paid approximately $12/hour for their participation (the rate of pay for each experiment differed slightly and was based on the payment recommendations of Prolific at the time of the study). The study was approved by the Michigan State University institutional review board, and all participants provided informed consent through an online form via Qualtrics (www.qualtrics.com). Prolific users who self-reported normal or corrected-to-normal vision and hearing, had English fluency, were between 18 and 65 years of age, had more than 10 previous submissions on Prolific, had a Prolific approval rating above 95, and had not participated in previous iterations of our study on Prolific were eligible to participate in the study. Otherwise, no restrictions were imposed on participation. Participants were instructed to complete the experiment in a single sitting using a desktop or laptop computer. There was no direct control of participant environment and behavior because the study was administered online, but participants were instructed to sit at their normal viewing distance and to avoid large movements during the study. Before the study began, participants completed a blind-spot identification procedure and a bank card scaling procedure (Brascamp, 2021) so that we could estimate viewing distance, as well as the size and aspect ratio of the pixels. Using this information, we displayed stimuli so that they would appear square and subtend approximately 20 degrees of visual angle from the participant's viewpoint.

### General procedure

We performed a series of three experiments, each building on the previous, to investigate the memory representations involved in slow change blindness. In each experiment, each participant completed a single critical trial where they judged whether the color of a cued area was the same or different as it was before a mask. Observers were presented with a 20-second long video in which a large, central, region underwent a slow color change over the span of the central 16 seconds. Immediately after the video finished, the scene was covered up by a mask, and the relevant screen region was cued with a single-word audio file. We then presented a comparison scene that matched a frame that had occurred at some time point during the slowly changing scene, such as the initial or final frame (Experiments 1 and 2) or an intermediate frame (Experiment 2). The observer’s task was to report whether the cued region in the comparison scene matched what they saw immediately before the image was covered. Unbeknownst to our observers, the cued area was always the large colored region that had undergone a color change during the preceding video.

In addition to the slow color change, these videos each contained three quick changes that happened over 1 second and were relatively easy to notice. These quick changes were included to impress upon the participant the fact that the scene was not static so that it could not be assumed that whatever was seen early on would remain that way, meaning that the comparison image really needed to be compared to the state that immediately preceded the blank. These videos were generated by the present authors using a novel, semi-automatic procedure (Frey et al., 2024) and are available online for use in studies such as this. Our previous work indicates that slow change blindness is not notably affected by the presence of such quick changes. When the number of such changes could vary between one and three, this number did not influence the observer's ability to detect the slow change (*r*^2^ = 0.0266) (Frey et al., 2024). In fact, that work also showed that observers performed similarly on the slow change detection task even in the absence of any quick changes.

**Figure 1.**
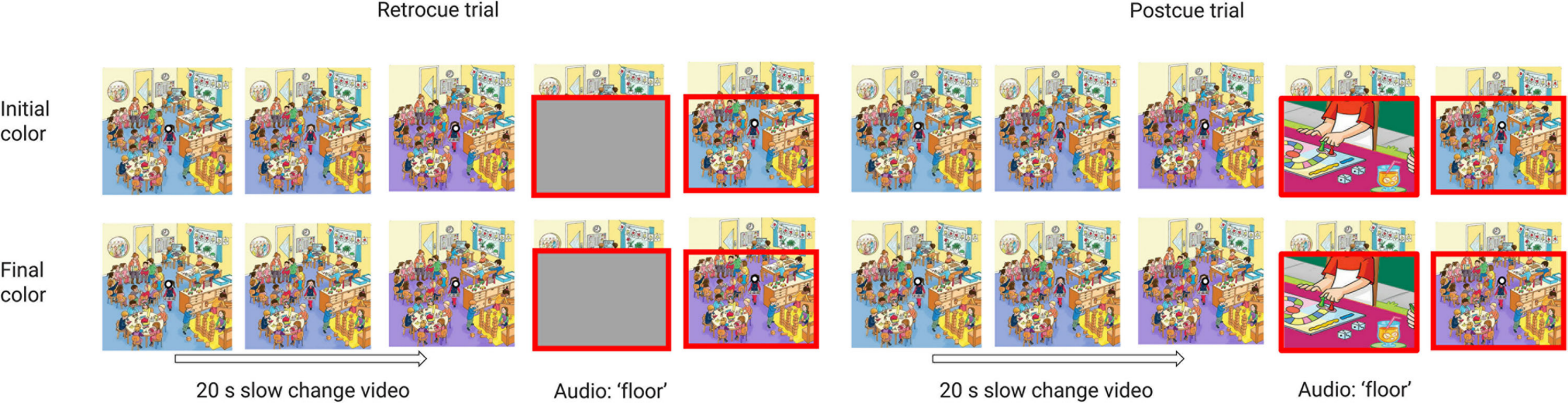
A schematic of a slow change trial in Experiment 1. In a slow change trial, a slowly changing stimulus was presented to the observer before the cover. As in the training, a retrocue trial involved preserving visual information with a gray box but a postcue trial overwrote visual information with a new image. After the cover, observers were presented with a frame that matched either the initial morph color or the ending morph color.

Prior to the trial, participants were instructed to fixate on a black-and-white circle at the center of the image at both the beginning and the end of the 20-second presentation. However, the circle disappeared after 2 seconds and did not reappear until 18 seconds into the presentation, and participants were free to look around the image during the absence of the circle. When the circle reappeared, a tone was played via the speakers or headphones as a reminder that the participant should return their gaze to the circle at the center of the image for the remainder of the trial. Observers were told that, during the 20-second viewing period, certain features of the image might change and that their task was to report via keypress whether the covered-up area was the same or different as it was *immediately* before the image was covered. The image shown after the mask was removed matched the final frame in terms of which quick changes had occurred, and the large color area matched a color that had been shown at some point during the slow change sequence. In Experiment 1, this was either the initial color or the final color of that area during the video (Figure 1). In later experiments, we also included comparison frames in which the large area had either a color that was intermediate between this initial and final color or a color that was substantially different from any color shown during the slow change sequence.

Each experiment included two conditions that differed in which type of memory stores we aimed to probe: short-lasting visual memory traces or longer term memory representations. To probe the former, we covered up the area of interest with a gray mask, which drew attention to the relevant screen location but with minimal visual disruption. Given that short-lasting visual memory traces are thought to be formed (and overwritten) any time visual input is present (Becker, Pashler, & Anstis, 2000; Landman, Spekreijse, & Lamme, 2003; Sligte, Scholte, & Lamme, 2008), minimizing visual disruption is critical here. We refer to this condition as the *retrocue* condition. In general, retrocues are spatial markers that are designed to cue an observer to relevant visual information after the information has been removed from the screen but before any disrupting visual information is presented (Griffin & Nobre, 2003; Landman et al., 2003; Sligte et al., 2008). In our Experiment 1, the temporal sequence was slightly different from what is described in this general definition of retrocues, because the mask acts both to remove the relevant visual information and simultaneously to cue the participant to where that information was (the location and extent of the mask indicate this). Still, we refer to this condition as the retrocue condition because the cue is introduced before any new visual information is introduced.

To probe longer term memory traces, we covered up the area of interest with a full-color and visually rich cartoon image mask, which drew attention to the relevant screen location while also providing substantial visual disruption. The visual disruption was intended to interfere with the storage and maintenance of fleeting visual memory traces, leaving only more robust and persistent traces for the participant to base their response on. We refer to this condition as the *postcue* condition. In general, postcues in this context are spatial markers that are designed to cue an observer to relevant visual information, after the information has been removed from the screen and also after disrupting visual information has been presented (Astle, Summerfield, Griffin, & Nobre, 2012; Sligte et al., 2008). In our Experiment 1, the temporal sequence was again slightly different because the cartoon cover does three things simultaneously: It removes the original visual content, it cues participants to the relevant scene region, and it also interferes with visual memory traces. We still use the term “postcue” because the cue does not precede visual disruption. In Experiment 2, we directly addressed some of the considerations concerning the sequence of events (see below).

In both conditions, the cue appeared at the same time and consisted of the mask (either blank or cartooned) with a red outline, as well as an audio file that played a one-word reference to the item in question (see below). Aside from the content of the mask (minimal visual information vs. an abundance of new visual information), the two conditions were the same.

**Figure 2.**
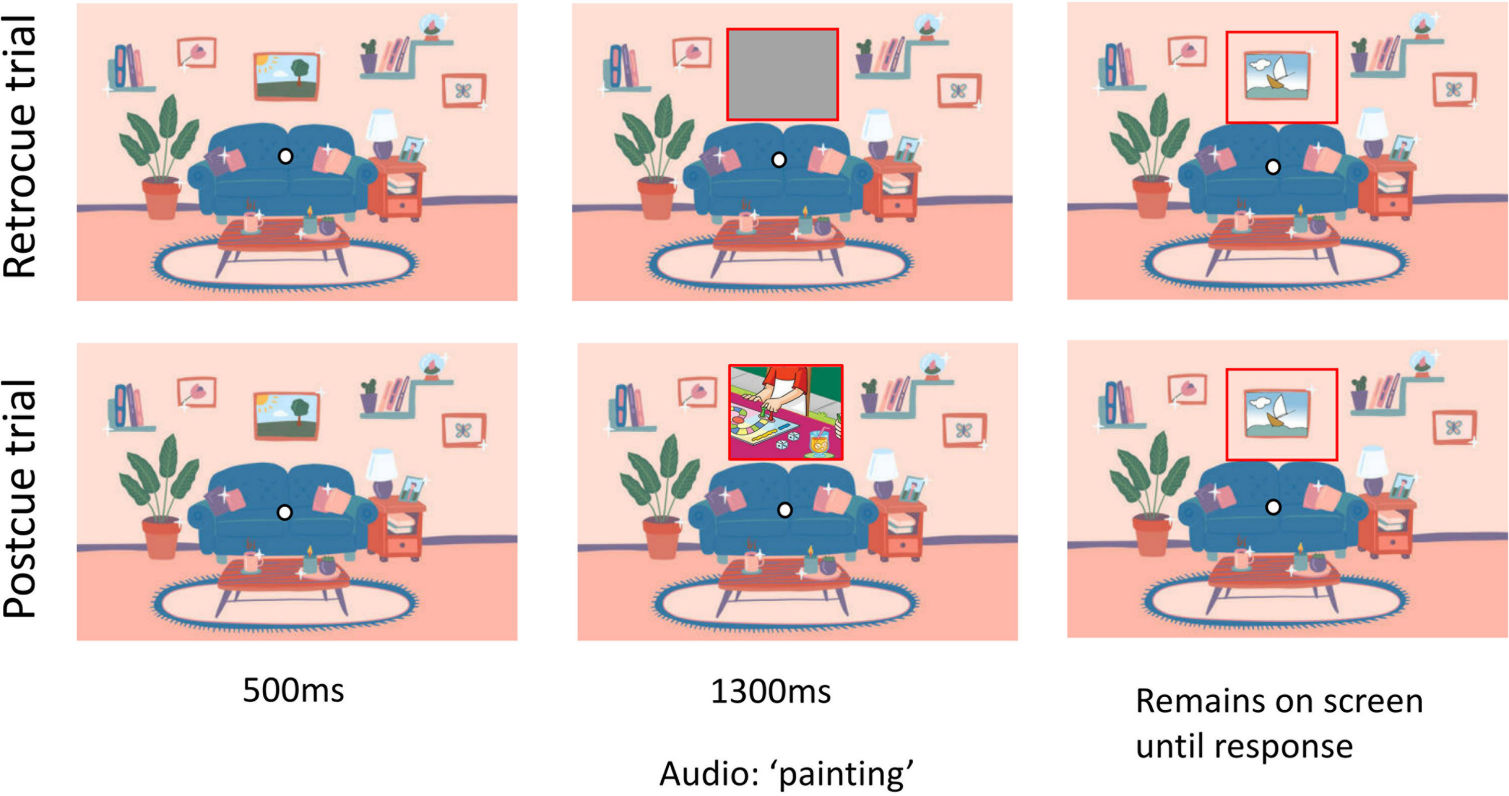
A schematic of a training trial (details differ between experiments, as described below). The top row depicts an example retrocue trial (visual information was preserved with a gray box) and the bottom row depicts an example postcue trial (visual information was overwritten with new information). See text for details.

Because visual memory traces can be very short lived (Sperling, 1960), in each experiment we took care to extensively train participants in quickly accessing their memory upon the presentation of a cue. Accordingly, the critical trial in each experiment was preceded by 100 training trials in which participants reported whether a cued area was the same or different between two still images. These practice trials helped participants learn to expect a cue and practice accessing memory quickly based on the cue. In each training trial, participants fixated on a central point. An image appeared for 500 ms, after which a portion of the image was covered up by a mask outlined in red. In addition to the location of the mask itself, a single-word audio file played simultaneously to further help participants direct their attention to the relevant, and covered-up, item while maintaining fixation at the center dot (Figure 2). Participants were told that they would be comparing an image before and after a portion of it was covered up and that they would be asked whether the image was the same or different. They were instructed to try to remember what was present behind the covered area as soon as the cover appeared and the audio file played. We added the audio cue because the rectangles, although certainly cueing attention to the relevant section of the image, often covered up other elements of the image in addition to the element that the participant would be asked about. In such cases, the audio file could help participants pay attention to the relevant aspect of the memory trace more quickly. The nature of the cover-up and the timing of the cue were different depending on whether the trial was a postcue or a retrocue trial and which experiment the observer was participating in (details of the cueing paradigm for each experiment are described below in their respective sections). After 1300 ms, the cover was removed, and participants responded with a keypress to convey whether the cued part of the image was the same as or different from what it was before it had been covered. The image remained on the screen until the participant entered their response.

The 100 training images were evenly divided between retrocue trials and postcue trials and between change trials and no-change trials. Moreover, for both cue types, 20% of the change trials involved a change in color between the image before the covering rectangle and the image afterward, and the remaining 80% of trials involved a different change, such as a change in the identity of an object or an increase or decrease in number of a certain object. This explicit separation of “color” and “other” changes allowed us to verify the participants’ performance on a task similar to that of the critical trial that followed the training, without making explicit that color changes would be relevant in the critical trial. The changes were created by editing one component of a scene using Photoshop 24.1.1 (Adobe, San Jose, CA) to produce two versions. We counterbalanced which 50 images were used in retrocue trials and which 50 images were used in postcue trials across two participant groups. The images were presented in a series of five blocks, each made up of 20 images presented in a random order. The proportion of 20% color change images was preserved within each block. At the end of each block of 20 training trials, participants were given general feedback about their performance, as well as a reminder of the instruction to mentally picture the area covered up as soon as it was covered.

Empty or incomplete data files, as well as data from people whose bank card scaling procedure was not consistent with square pixels (indicating that they did not complete the scaling procedure well and as a result stimuli were not presented as intended) were excluded from analysis.

### Statistical analysis

Given that the data collected in these studies were binomial (responses were either “same” or “different” and each participant was presented with a single trial and provided a single response), some common statistical tools (such as analyses of variance and *t*-tests) were not appropriate. Instead, we fit a generalized linear mixed model with the form same ∼ cue type * comparison color + (1/*image*) to our data with “binomial” as the family. This formula describes the hypothesis that the response variable *same* (0 or 1) depends on the fixed effects of cue type (retrocue or postcue), comparison color (e.g., initial or final), and their interaction, and on the random effect of image (three possible images). Cue type, comparison color, and their interaction are fixed effects because they are assumed to affect the response variable in a systematic way, and we tested all levels that we cared about. Image is a random effect because the specific image that people saw could affect the response variable, yet the subset of images used in the study is not an exhaustive list of images. To evaluate the significance of the factors of the model, the R drop1() method with (test = “Chisq”) (R Foundation for Statistical Computing, Vienna, Austria) was used to compute a likelihood ratio test (LRT) by dropping one factor at a time. Respecting the principle of marginality, this function only makes a comparison between the full model (above) and an identical model that does not include the interaction term *same ∼ cue type + comparison color +* (1/*image*), so it provides an estimate of the significance of the interaction.

Although the above-described model is suitable for comparing many conditions simultaneously (e.g., we apply it to the data from two cue types and three comparison colors at once), we were also interested in some pairwise comparisons between conditions, specifically between conditions that shared the same cue type but differed in comparison color. In those cases, we used a simpler linear mixed model of the form *same ∼ comparison color +* (1/*image*) and included data only from the two conditions we wished to compare. Again, significance was tested by computing likelihood ratios, now between the full model and the same model without the factor “comparison factor.” In other words, these pairwise tests were used to determine whether there was a difference in “same” responses between two specific comparison colors within a cue type.

## Experiment 1

In Experiment 1, 205 participants were randomly assigned to one of three slow change videos (each with three quick changes) and to one of four conditions: retrocue initial, retrocue final, postcue initial, or postcue final. “Initial” and “final” here refer to whether the comparison image matched the initial video frame or the final video frame with regard to the slowly changing area. Of the three slow change videos used in this experiment, two involved a slow color morph from yellow to orange and the third involved a slow color morph from blue to purple. The cueing procedure used in both the training trials and the slow change trial was the same. For the retrocue conditions, the relevant image part was covered up with a gray box outlined in red; for the postcue conditions, the part was covered up with a box containing a randomly oriented cropped portion of a different full color cartoon image, also outlined in red. In both conditions, a brief audio file played to further direct attention to the colored region, and the red outline remained on the screen after the cover was removed. Figure 1 shows a schematic of a slow change trial.

**Figure 3.**
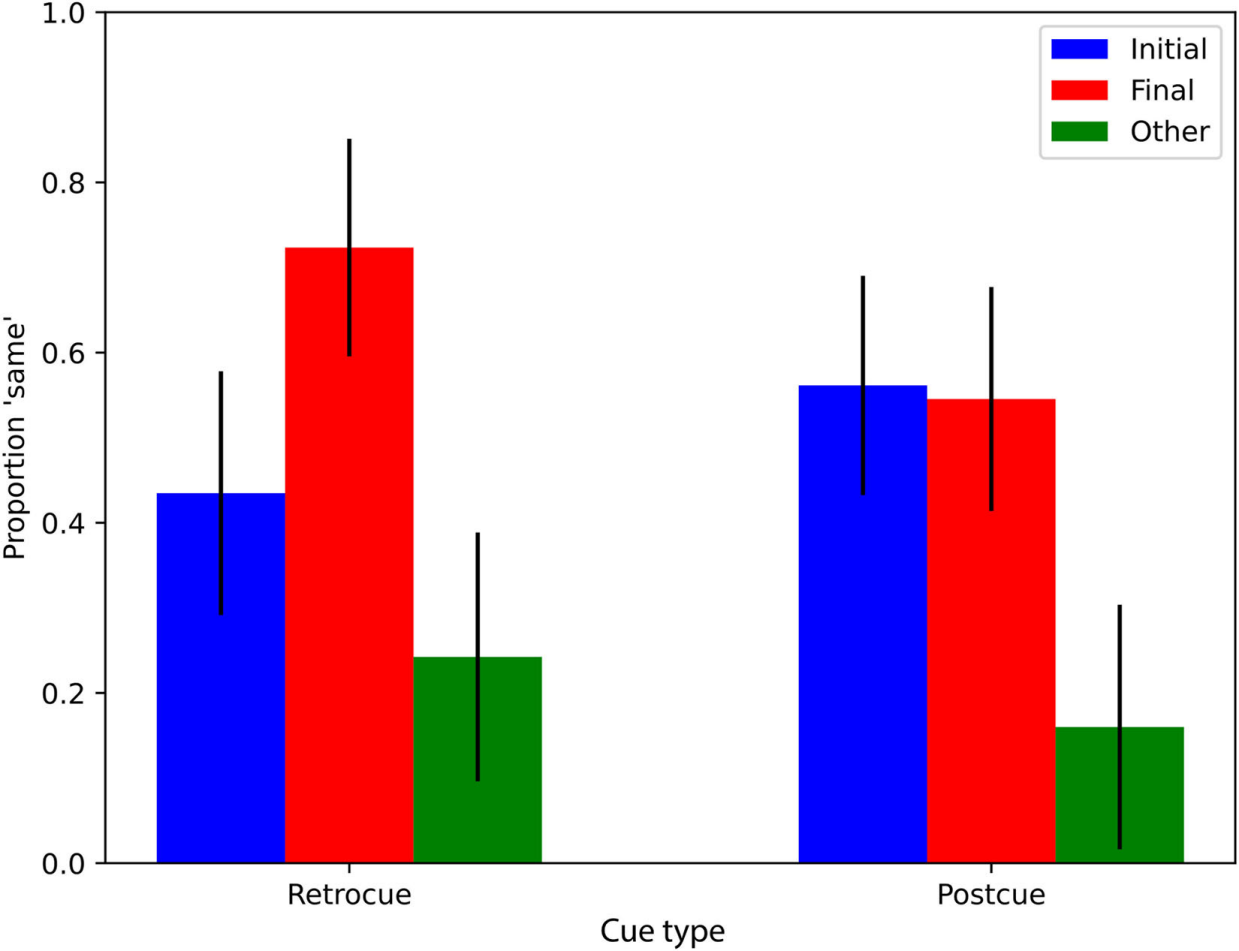
Results of Experiment 1a (blue and red bars) showing the proportion of participants who responded that the comparison image was the same as the image immediately before the covering rectangle appeared. Each bar corresponds to a different group of observers. Black bars represent 95% binomial confidence intervals. The green bars reflect the results of follow-up Experiment 1b.

We plotted the average proportion of “same” responses across participants in each condition (Figure 3, blue and red bars). Black bars represent 95% binomial confidence intervals. The full generalized linear mixed model (see Statistical analysis) fits the data significantly better than a model without the interaction between cue type and comparison color (LRT = 5.5598, *p* = 0.018), indicating that the interaction is significant. This is consistent with the impression from the plot, as participants overall tend to say “same” more when the comparison color matches that in the final frame (red bars) than when it matches the initial frame (blue bars), but this tendency depends markedly on the type of cue. Specifically, following retrocues, participants respond “same” significantly more often when the comparison color is the final color versus the initial color. Indeed, when comparing specifically the retrocue initial and retrocue final conditions, a generalized linear mixed model with comparison color as a fixed effect explains the data significantly better than a model without that factor (LRT = 9.31, *p* = 0.0023). Following postcues, these proportions are much more even between comparison colors, and, indeed, when comparing specifically the postcue initial and postcue final conditions, a generalized linear mixed model with comparison color as a fixed effect does not explain the data better than a model without that factor (LRT = 0.009, *p* = 0.923). These results are consistent with short-lived visual memory traces being preserved in the retrocue condition and mostly reflecting the final color, such that, following a retrocue, participants say “same” more when the comparison color matches this final color. At the same time, the lack of response difference between comparison colors in the postcue condition seems to suggest that longer term memory stores (which are those that remain after short-lived visual memory is overwritten) contain relatively similar amounts of evidence for both the initial color and final color.

However, the results of Experiment 1 allow for the possibility that participants did not draw on any memory representation in the postcue conditions and that the proportions of “same” near 0.5 in those conditions are a result of guessing. Before drawing any strong conclusions, therefore, we performed a brief extension of Experiment 1 with 58 participants where the comparison frame was an “other” color: a color that was neither the initial color nor the final color of the slow change video but something substantially different. This condition was added as a control: If participants truly had no memory representation of the color of the cued object, then the proportion of “same” responses in the “other” color condition should be similar to that of the initial and final conditions. However, if there is a difference then this would confirm that participants are not guessing and that they have some memory of the colors presented during the slow change video. The three slow change videos used were the same as in Experiment 1 (from here on, we will refer to the original Experiment 1 as 1a and the extension as 1b). For the yellow–orange morphs, the “other” color was purple in one case and blue in the other, and for the blue–purple morph the “other” color was orange. Experiment 1b was identical to Experiment 1a in all other ways. Consistent with the idea that observers were not merely guessing, we found that, when an “other” color frame was presented for comparison, participants were not likely to report “same” (green bars in Figure 3). Four pairwise tests (see Statistical analysis) were performed to determine whether the proportion of “same” responses was significantly different in the “other” color condition as compared to the initial or final conditions within each cue type condition. A generalized linear mixed model including the fixed effect of comparison color was significantly better at explaining the data compared with a model without comparison color as a fixed effect when the models were applied to the data from the conditions postcue final and postcue other (LRT = 13.154, *p* < 0.001), when they were applied to the postcue initial and postcue other data (LRT = 14.376, *p* < 0.001), and when they were applied to the retrocue final and retrocue other (LRT = 19.09, *p* < 0.001). When applied to the data of the retrocue initial and retrocue other conditions, the full model was only marginally better at explaining the data (LRT = 3.45, *p* = 0.06). Of course, these results do not rule out the possibility of guessing during this experiment. There certainly may have been participants who did not form a strong representation and had to guess. This, in combination with the possibility of response biases, means that the absolute values of the bars plotted should be interpreted with caution. Notwithstanding, differences between the bars can be interpreted confidently because any effect of guessing would be uniform across the conditions. The lower proportion of “same” responses for the “other” color comparison frames is evidence that participants had *some* representation of the color presented during the video and were not uniformly guessing. As such, these data show that participants maintained some representation of the slow change target in both cue conditions.

## Experiment 2

**Figure 4.**
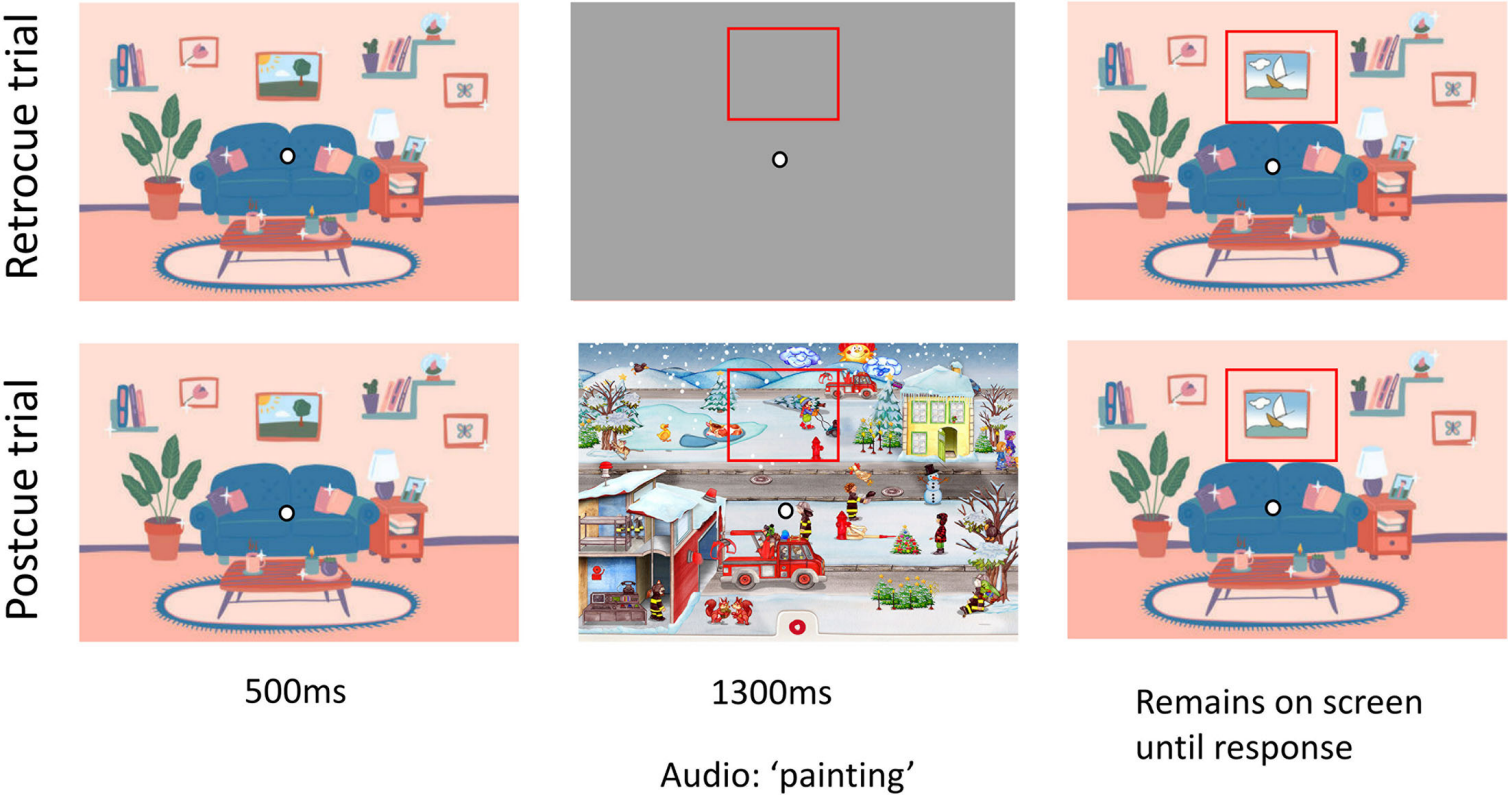
In the Experiment 2 training trials, the cover was not restricted to the area of change. Instead, we introduced a full screen mask with a smaller red outline to cue the area of interest.

**Figure 5.**
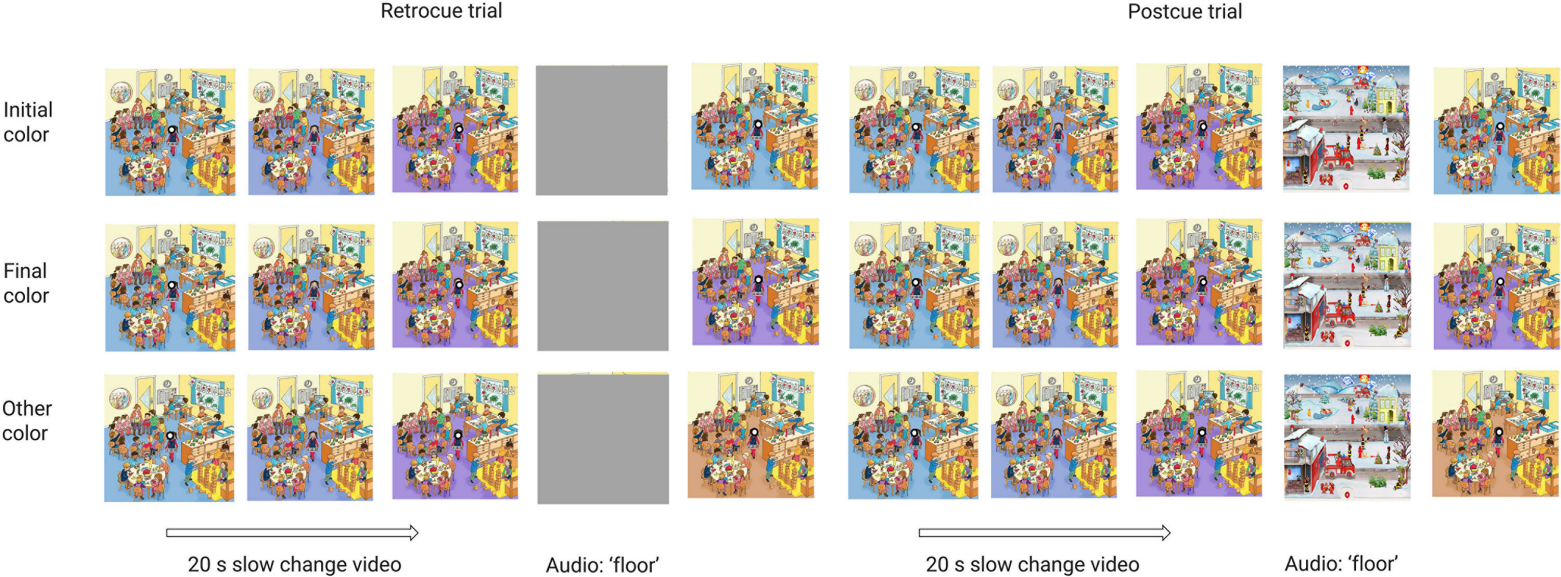
In the critical trial in Experiment 2, the cover was not restricted to the area of change in the slow change trial. The mask covered the entire image. Given that the area of change was large and the audio cue was detailed enough, we did not include a red box to further cue the area of change.

Experiments 1a and 1b together provide reasonably compelling evidence that there are multiple types of memory traces formed during slow change blindness. The short-lived visual memory traces (captured by performance in retrocue trials) result in a bias toward the final color, whereas more persistent memory traces (operationalized by performance in postcue trials) do not show this trend. Before further exploring the nature of assumed memory traces involved, we wished to gain more confidence that the two cuing paradigms neatly separated different memory systems. Specifically, we wished to ensure that short-lived visual memory traces played no role in the postcue condition. Here, we considered the issue of temporal sequence that we first addressed above in the General procedure section: In the postcue condition of Experiment 1, the cover acts simultaneously to remove relevant visual information from the screen, to cue the participant to the relevant screen region, and also to disrupt short-lived visual memory traces. We wondered whether this might enable participants to query short-lived visual memory traces after all, right before they were disrupted. Of note, existing work using postcues typically features first a mask that disrupts short-lived traces and only then a cue that directs the participant's attention (Astle et al., 2012; Sligte et al., 2008). To address this potential concern, we performed a second experiment, in which we covered the entire image rather than just the area of interest (in both the training and final trials; see Figure 4 and Figure 5, respectively). This means that the cover, although still simultaneously removing relevant visual information from the screen and, in the postcue condition, disrupting short-lived visual memory traces, no longer acted to cue the participant to any specific part of the scene. In the retrocue condition this variant of our design involved a gray rectangle matching the size of the entire image and the shade of the background (i.e., the image just disappeared). In the postcue condition, the image was covered with a complete full-color cartoon image placed either right way up or inverted. For the slow change trial, we also eliminated the red outline that had appeared at the same moment as the mask in Experiment 1. This was possible because the slow changing colored area was sufficiently large for the audio cue on its own to inform participants which scene element the same/different task pertained to (e.g., the entire floor of a room). In the training trials, in which changes could be confined to much smaller scene elements, we did stick to using the red outline to highlight the relevant screen region (Figure 4). A practical obstacle here was that, in the postcue condition, the red outline was difficult to see when it appeared at the same time as the now larger covering image. Accordingly, in Experiment 2, we delayed the outline until 100 ms after the cover's appearance, for both cue conditions. This delay was sufficient to allow an apparent visual transient of the red outline while still appearing simultaneous to the observer, and it aligns our condition more closely with that of classic postcues (Astle et al., 2012; Sligte et al., 2008).

In addition to these changes, we doubled the number of distinct slow change videos we used in the experiment from three to six in order to evaluate the generality of our findings. The 417 participants were randomly assigned to one of the six slow change videos and to one of six conditions: retrocue initial, retrocue final, retrocue other, postcue initial, postcue final, or postcue other.

We also expanded on Experiment 1 by explicitly verifying whether the participants noticed the slow color change. In Experiment 1, we had assumed the presence of change blindness based on previous work using similar versions of these videos, which indicated that the change detection performance was below 2% across over 1000 participants (Frey et al., 2024), but here we opted to assess change blindness directly. Accordingly, after inputting their same/different responses, we asked participants whether they had noticed any changes in the image during the 20-second video that preceded the covering rectangle. If they responded “yes,” they were presented with a textbox to freely describe any changes they had noticed. Recall that participants had been explicitly informed that some things might change during those 20 seconds, and recall that there were three quick changes in addition to the slow color change.

Participant responses to the free report of changes were manually coded to determine which changes had been reported. Any response that mentioned a change in the element that underwent the color change, even if color was not explicitly mentioned, was marked as an instance of “noticing” the slow change. Although they were asked specifically about changes during the 20-second viewing period, some individuals gave ambiguous descriptions such that we could not tell whether they were describing the slow color change or the “change” that happened during the cover up (i.e., the color comparison they were asked about in the same/different task). To be conservative, these responses were also marked as noticing the slow color change. Based on these criteria, out of 417 participants, we excluded seven participants who noticed the change and seven participants who ambiguously described the change—an overall proportion of 0.034. These individuals were excluded from analysis. The remaining 96.6% of participants were classified as being blind to the slow change.

**Figure 6.**
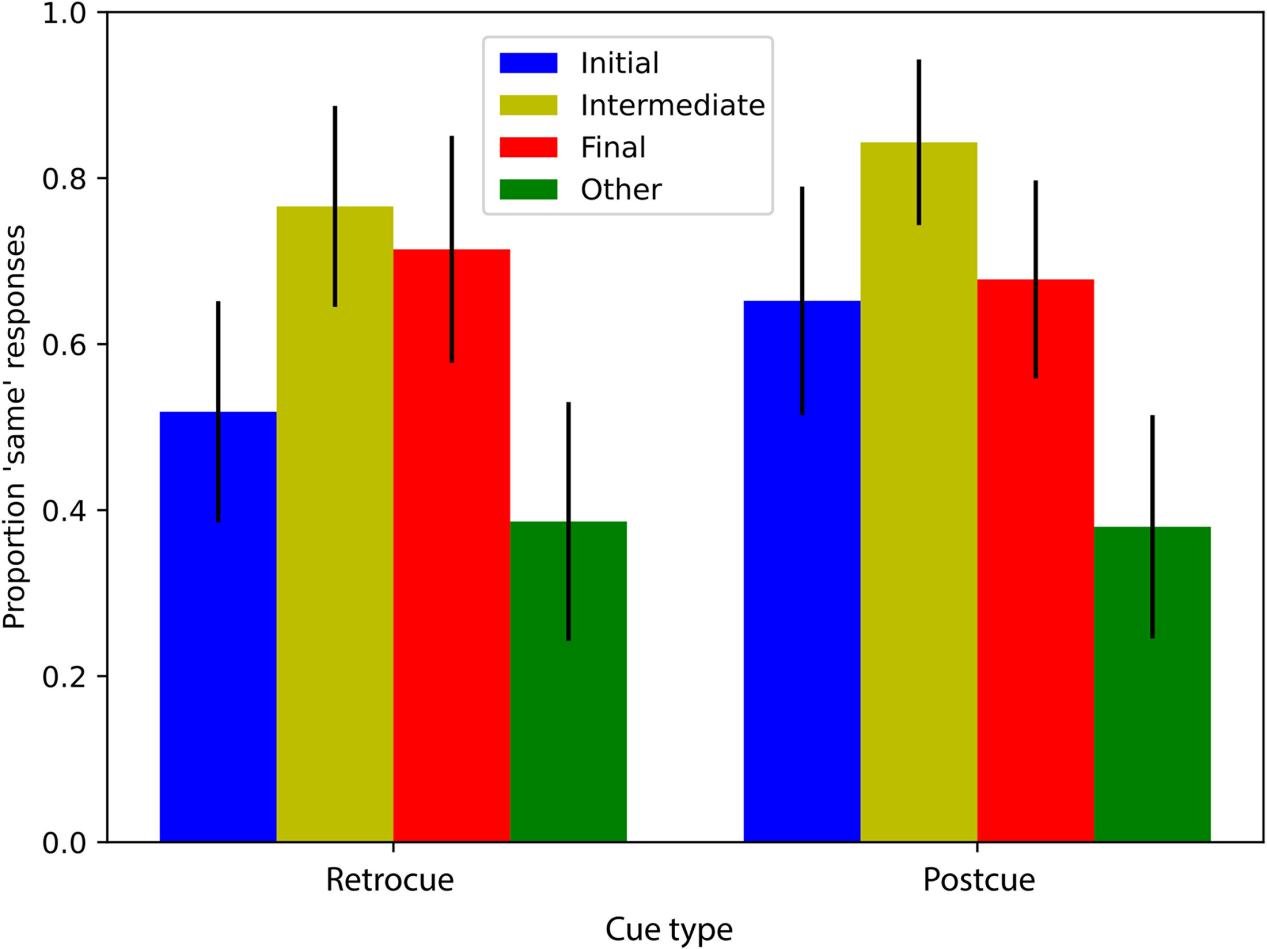
The proportion of participants, in each of the six conditions of Experiment 2 (blue, red, and green bars) and the two conditions of the Experiment 2 follow-up (yellow bars), who responded that the comparison image was the same as the image immediately before the covering rectangle appeared. Each bar corresponds to a different group of observers. Black bars represent 95% binomial confidence intervals.

Figure 6 (blue, red, and green bars) shows results of the same/different task. Visual inspection of the plot suggests a data pattern that is very similar to that of Experiment 1. First, the proportion of “same” responses is lower in the “other” color conditions than in the initial and final conditions, indicating that participants were not merely guessing. We do note that the proportion of “same” response in the “other” color condition was higher than it was in Experiment 1. This might suggest that the different mask design in Experiment 2 degrades memory representations and thereby renders the task more difficult. However, a generalized linear mixed model of the data from both Experiments 1 and 2 (excluding the “other” color conditions) together with mask type as an additional fixed effect (this was the only difference between the two experiments; formula: same ∼ cue type * comparison color + mask + [1/*image*]) did not explain the data better than a model without mask as a fixed effect (LRT = 2.56, *p* = 0.109). This indicated that the type of mask (localized box or full screen) did not influence participant responses to the initial and final comparison frames in a systematic way. Furthermore, within Experiment 2, a generalized linear mixed model applied to a single initial or final comparison color condition, as well as the corresponding “other” condition, invariably explains the data better when it does include comparison color as a fixed effect than when it does not: postcue final and postcue other (LRT = 10.15, *p* = 0.0014), postcue initial and postcue other (LRT = 10.75, *p* = 0.0010), retrocue final and retrocue other (LRT = 15.66, *p* < 0.001), or retrocue initial and retrocue other (LRT = 4.91, *p* = 0.027). This finding again indicates that participants are not uniformly guessing but rather that their memory representations provide more evidence for the initial and final color than for the other color. In agreement with Experiment 1, Figure 6 additionally suggests that, for the retrocue condition, the proportion of “same” responses is higher for the final color than for the initial color. Indeed, a generalized linear mixed model applied to the retrocue initial and retrocue final condition pair explains the data significantly better when it includes comparison color as a fixed effect than when it does not (LRT = 4.55, *p* = 0.0328). Also in agreement with Experiment 1, Figure 6 suggests that this difference between the initial and final color was absent in the postcue condition, which would suggest an interaction between cue type (retro or post) and comparison color (initial or final). The difference is indeed absent in the postcue condition (generalized linear mixed model applied to the postcue initial and postcue final condition pair yields LRT = 0.006, *p* = 0.937), but this impression of an interaction is not supported by statistical analysis in Experiment 2 as it was for Experiment 1. This time, the full generalized linear mixed model applied to the data from the initial and final conditions and both cue conditions does not explain the data significantly better than a model without the interaction (LRT = 2.37, *p* = 0.124).

Faced with these similar proportions of “same” responses for the initial and final comparison frames in the postcue conditions of both Experiment 1 and Experiment 2, we decided to extend Experiment 2 with an additional condition to distinguish between potential explanations for this data pattern. In particular, it is possible that memory representations in the postcue condition specifically contain evidence for the initial and final color of the video, akin to classic primacy and recency effects in memory (Postman & Phillips, 1965). Alternatively, it is possible that the postcue condition probes a memory representation that is relatively general and is consistent with various colors that resemble the initial and final one (even if, evidently, it does not form a good match with the “other” color). Such a non-specific representation could correspond to a spectrum of colors ranging from the initial to the final color, to which an intermediate color might form a particularly good match. Primacy and recency effects would not apply to such an intermediate color. To further investigate this, we conducted a brief extension of Experiment 2 with 126 participants in which we presented an intermediate frame (generated by taking the average of the initial and final comparison frames, which is equivalent to the frame that was presented midway in the slow change video) as the comparison color in both the retrocue and postcue conditions. The result, shown as yellow bars in Figure 6, is inconsistent with an explanation centered on primacy and recency; for both cue conditions, the proportion of “same” responses is high for the intermediate color. A generalized linear mixed model applied to the retrocue final and intermediate conditions (LRT = 0.039, *p* = 0.844), postcue initial and intermediate conditions (LRT = 2.59, *p* = 0.108), and then the postcue final and intermediate conditions (LRT = 2.84, *p* = 0.092) does not explain the data significantly better when it includes comparison color as a fixed effect. These results indicate that the proportion of “same” responses to the intermediate color only numerically exceeds the proportion of “same” responses to the final color and the proportion of “same” responses to the initial color in the postcue condition. However, a generalized linear mixed model applied to the retrocue initial and intermediate conditions does explain the data significantly better when it includes comparison color as a fixed effect, indicating that, within the retrocue conditions, the proportion of “same” responses to the intermediate color is statistically greater than the proportion of “same” responses to the initial color. This higher proportion of “same” responses following a retrocue in the intermediate condition points to the possibility that there is some influence of non-final states on the later representations. The similar proportions of “same” responses to the initial and final frames in the postcue condition, on the other hand, may indeed indicate that the long-term memory trace that participants rely on following a postcue is relatively non-specific and matches the spectrum of colors ranging from the initial to final colors presented during the video.

## Experiment 3

The results from Experiments 1 and 2 seem to suggest that two distinct memory representations were in play during our experiment. First, during the retrocue trials, participants could draw on a relatively precise, but short-lasting and fragile (iconic) memory representation of what occurred recently during a slow-change video. This representation is responsible for the relatively high proportion of “same” responses for the final color comparison frames during those trials, as well to the low proportion of “same” responses for the initial color comparison during those trials (the representation is precise enough not to match that initial color). Second, during postcue trials, in which the mask interferes with this precise memory representation, participants relied on a relatively coarse, but more stable representation consistent with a range of colors resembling those shown during the video, and they could reliably reject the “other” color. This explains why, during those trials, the proportions of “same” responses were similar for the initial, intermediate, and final comparison frames yet consistently higher than for the “other” comparison frames that involved a color from the other side of the color wheel.

Experiment 3 was designed to test two hypotheses that emerged from this impression. This experiment had a structure identical to Experiment 2, except that it involved 20-second videos in which the color of the target object did *not* change. Specifically, each video used in Experiment 3 was identical to each video used in Experiment 2, but throughout its duration had the color that, in Experiment 2, had been the *final* color. The comparison frames used in Experiment 3 were as they were before. That is, for half of the participants, the comparison frame matched the frame that had been the final frame in Experiment 2 and, therefore, the same frame that was present throughout the 20-second video in Experiment 3. We refer to this comparison condition as *final/present throughout*. On the other half of the trials, the color of the comparison frame matched that of the frame that had been the initial frame in Experiment 2. This frame therefore never appeared during the video of Experiment 3, but its color was from a similar region of the color wheel as the color present in the video. We refer to this comparison condition as *initial/never present*. The hypotheses addressed by the experiment are as follows. First, we have shown that, during retrocue trials, participants rely primarily on a relatively precise representation of recent colors. This should lead to a high proportion of “same” responses in the final/present throughout condition and a low proportion in the initial/never present condition. This difference between the final/present throughout and initial/never present conditions may be even more extreme than the corresponding difference between the final and initial conditions of Experiment 2 if there does happen to be some influence of earlier information in the fragile short-term representation (i.e., we would expect the performance difference between final and initial colors to be larger in Experiment 3 than in Experiment 2). Second, we have shown that, during postcue trials, participants rely primarily on a relatively coarse representation that is consistent with a range of colors that resemble those shown before. The coarse nature of the color representation could mean that the final/present throughout and initial/never present conditions may yield similar proportions of “same” due to the similarity of the colors, despite the fact that only one color was involved in the target stimulus. This prediction may be somewhat surprising, because the initial/never present color was never shown, but it is consistent with the explanation that the memory representation used in postcue trials is coarse enough to match similar colors even if they were not seen during the video.

In this experiment, 308 naïve individuals participated and were randomly assigned to one of the six no-change videos and one of the four groups: retrocue initial/never present, retrocue final/present throughout, postcue initial/never present, and postcue final/present throughout.

**Figure 7.**
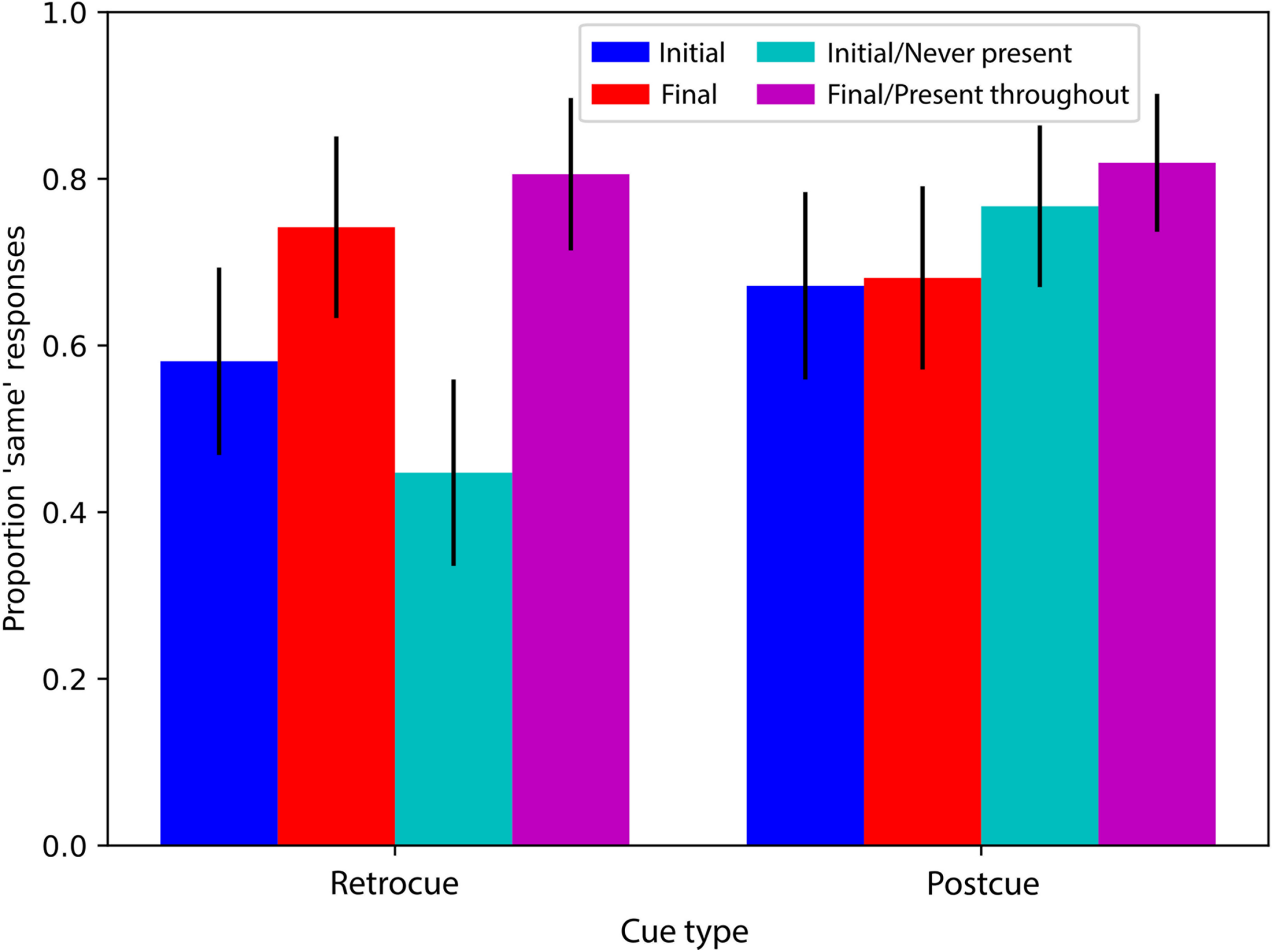
The proportion of participants who responded that the comparison image was the same as the image immediately before the covering rectangle appeared. Each bar corresponds to a different group of observers. The blue and red bars are from Experiment 2, the same data as shown in Figure 6. The cyan and magenta bars are the results from Experiment 3. Black bars represent 95% binomial confidence intervals.

Participant responses to the free report of changes were manually coded to determine which changes were reported. Given that there was no slow color change, we did not expect any responses describing this change and in fact did not receive any. Figure 7 shows results of Experiment 3 (cyan and magenta bars) next to the replotted results of Experiment 2 (the blue and red bars, replotted from Figure 6).

The results of the retrocue condition match the hypothesis articulated above: There was a lower proportion of “same” responses for the initial/never present condition than for the final/present throughout condition. A generalized linear mixed model considering these two conditions explains the data significantly better when comparison color is included as a factor than without it (LRT = 23.56, *p* < 0.001). Numerically, it seems that this difference is more pronounced than the corresponding difference between the initial and final conditions in Experiment 2, but this impression is not supported by statistics (a generalized linear mixed model with factors experiment and comparison color does not explain the retrocue data significantly better when it includes the interaction between those factors than when it does not; LRT = 2.84, *p* = 0.092). Notably, the proportion of “same” responses in the retrocue initial/never present condition of Experiment 3 (left cyan bar) is numerically as low as that proportion was in the “other” color condition of Experiment 2 (Figure 6), confirming that the representation used in the retrocue task is sufficiently precise to distinguish between a color shown in the videos of Experiment 3 and a relatively similar color that was never shown.

The results of the postcue condition also match the hypothesis articulated above: The proportions of “same” responses in the initial/never present condition and the final/present throughout condition are similar and not significantly different (a generalized linear mixed model comparing the initial/never present and final/present throughout comparison frames of the postcue condition does not explain the data better when it includes comparison color as an effect than when it does not; LRT = 0.647, *p* = 0.421). Compared with Experiment 2, the proportions of “same” responses were overall higher in Experiment 3, but their dependence on comparison color did not differ between experiments. Specifically, a generalized linear mixed model comparing the initial/never present and final/present throughout postcue conditions from Experiment 3, as well as the initial and final postcue conditions from Experiment 2, explains the data better when it includes experiment as a factor (LRT = 5.15, *p* = 0.023), but an interaction between experiment and comparison color does not further improve the model (LRT = 0.261, *p* = 0.609). These results are consistent with the notion that, in the postcue conditions, participants rely on a coarse representation that matches a range of colors *similar* to what was shown during the video (even if the comparison color was not actually present in the video). Nevertheless, we reiterate that the postcue condition yielded a significantly lower proportion of “same” responses when a color from the opposite side of the color wheel was used for comparison (the “other” color condition of Figure 6), indicating that the representation is not so coarse as to match any color. In other words, although the postcue results of Figure 7, on their own, could lead one to conclude that the postcueing procedure leaves no color memory whatsoever, resulting in similar proportions of “same” responses for all color conditions, such a conclusion would be inconsistent with the results of Figures 3 and 6, as it would not explain why those figures show such low proportions of “same” responses for the “other” color.

## General discussion

In this study, we have shown that, during slow change blindness, observers form two distinct types of representations of the changing object: a rich but fragile and fleeting representation of the object as it just was on the screen, and a coarse but stable representation that can match a variety of similar stimuli. As in classic change blindness, observers maintain information about different stimulus states even when they do not report that the stimulus has changed. However, the two representations formed either are too sparse (longer term stores) or decay too quickly (brief but fragile stores) such that even a comparison of the two would not necessarily lead to detection of the change. Therefore, we do not provide evidence for the failure to compare hypotheses (Laloyaux et al., 2008).

Our results are consistent with an averaging of stimulus information across time, as long as the averaged result is still relatively general. Serial dependence models are one type of models that describe an altering of information or an integration across time (Manassi, Murai, & Whitney, 2023; Pascucci et al., 2023), and have previously been applied to color judgments (Barbosa & Compte, 2020). The idea of averaging across time might predict that an intermediate frame would be the best match because it reflects the average color information across the video. The initial and final frames would still be close enough in color to this representation to elicit a “same” response. In other words, the high proportion of “same” responses to the intermediate frame in the postcue condition of Experiment 2 could also provide support for a representation that is an average of the visual information across time. However, given the high proportion of “same” responses to the “initial/never present” postcue condition in Experiment 3, as well as the relatively high proportions of “same” to the initial and final frames in the postcue condition of Experiment 2, we note that this representation is still coarse enough that it can be matched to a range of colors.

The tendency, in retrocue conditions, for “same” responses to a comparison frame that matched the final color is consistent with the implicit updating hypothesis: Fragile and fleeting visual information is constantly overwritten by new visual information (Hollingworth & Henderson, 2004; Suchow & Alvarez, 2011). As such, our results suggest that memory representations can be altered in the face of incoming visual information, without prompting an explicit comparison between what the representations held previously and the latest information (perhaps because the difference is below the threshold for drawing attention) (Hollingworth & Henderson, 2004). Having said that, our data do not distinguish whether the precise memory representation that drives behavior in our retrocue conditions is shaped exclusively by the very last video frame or by a slightly longer stimulus history. In the extension of Experiment 2, we found the proportion of “same” responses following a retrocue to be similar (and numerically higher) when the comparison image had an intermediate color compared with when it had the color of the final frame itself. This may mean that the memory trace involved is also shaped by frames that preceded the final video frame. The same impression arises from the fact that the retrocue condition yielded a numerically higher proportion of “same” responses in the final/present throughout condition of Experiment 3, where the final few frames all matched the final color, than in the final condition of Experiment 2, where the final few frames included the final color but also some more intermediate colors. This suggests that the memory trace may be formed over a few more recent frames rather than the final frame exclusively.

Similarly, in the postcue condition, the initial, intermediate, and final comparison frames all gave similar proportions of “same” responses, and, taken together, these results underscore the notion that it is often more useful to conceptualize memory representations as containing evidence for a range of colors (i.e., as a probability distribution across color space) (Chetverikov, Campana, & Kristjánsson, 2017; Kristjánsson, 2023) than as corresponding to any particular color. Throughout this manuscript, and in spite of the fact that our experiment only probed discrete colors, we have tried to use terminology that reflects such considerations, such as when describing a memory system as “being dominated by” or “containing information on” a given color, rather than as “corresponding to” that color. Nonetheless, this idea of memory representations containing evidence for a range of colors is consistent with our interpretation of the nature of color information available in longer term stores.

Our results do not provide strong evidence that earlier states of the slow-change video still leave a trace in memory representations by the time the observer gives their same/different response. Of particular relevance here is the observation, in Experiment 3, that the proportion of “same” responses to a comparison image with the initial color was statistically indistinguishable, regardless of whether this initial color was part of the preceding video initial or not initial/never present. Other work has shown such an influence of earlier states on memory following slow-change videos (Laloyaux et al., 2008). What may explain this difference? One potentially relevant consideration is the nature of the visual material; although we focused on color memory, that prior study examined memory of facial expression. Another factor that may be relevant is the way in which memory was probed, which may influence the memory system that an observer ends up using. Even in our postcue condition, our use of comparison images and same/different judgments may have invited a reliance on memory systems that are closely tied to visual, as opposed to semantic, content. Each comparison image followed immediately after the video and was presented in the same screen position, rendering a comparison in terms of low-level visual features potentially fruitful. The prior study, in contrast, relied on observers choosing a best match from an array of options that were spread across the screen, as well as on metacognitive confidence judgments about those choices. As such, their method might have probed different memory systems less closely tied to visual and more to semantic content.

In the above, we have remained mostly neutral regarding the specific memory systems involved in our task, beyond arguing that the cover in postcue conditions disrupted short-lived visual memory traces but the cover in retrocue conditions did not. Is it possible to be more specific about the exact memory systems involved? Short-lived visual memory systems that are affected by visual disruptions include, most famously, iconic memory (Sperling, 1960), but there is also evidence for a slightly longer lasting high-capacity visual store, termed fragile visual short-term memory (Sligte et al., 2008). Both of those systems are sensitive to disruption by new visual information, so both would likely contribute more strongly to reports in our retrocue condition than in our postcue condition, especially in Experiments 2 and 3, where we took greater care to preserve the traces in the retrocue condition and destroy them in the postcue condition. Candidate memory systems that remained after visual disruption in our postcue condition include working memory but also long-term memory, and it is conceivable that some part of the shorter lived visual memory traces survives visual disruption, as well. Based on the available evidence, no definitive statements about these issues are possible, except that both iconic memory and fragile visual short-term memory are likely candidates for explaining the difference between our postcue results and our retrocue results.

A related question, that we touched on in the introduction, is the central question that motivates interest in slow change blindness: Why is it that observers fail to notice this type of slow change to begin with? More specifically, in the present context, what role do the memory systems discussed above play in this failure? In our specific experiment, short-lived memory traces seem to be too fleeting to support a comparison between earlier and later stages of the change, whereas long-lived memory traces appear too coarse. So, it is completely possible that participants both form and access both types of memory traces during slow-change blindness, yet still miss the change. If this is true, then one testable prediction would be that slow change blindness should fail when the change in question is so substantial that the coarse, long-lived memory traces allow one to distinguish earlier and later stimulus states. For example, a slow change between what we have called the “other” color and what we have called the “final”’ color would not go unnoticed, given our present evidence that long-lived memory traces allow a distinction between those two colors. If, on the other hand, slow change blindness is robust, even for such colors, then that would argue in favor of the failure to compare hypothesis: Although the coarse memory representation could help identify the change, the relevant representations are not compared.

One noteworthy result of this study that we have not emphasized is that observers overwhelmingly failed to report the slow color change, *even after having been explicitly directed to the relevant aspects of the stimulus* in Experiment 2. Recall that, in that experiment, observers first performed the same/different task and then responded to the open question about the slow color change. Even though other studies have reported that people could “discover” information about the earlier state of a stimulus when prompted (Simons et al., 2002), the continued low change detection rate in our Experiment 2 underscores the robustness of the slow change blindness phenomenon even when prompted.

To our knowledge, this is the first study to use the specific approach to slow change research that we introduced in a previous paper (Frey et al., 2024). As detailed in that paper, the stimuli used in this approach are generated such that experimenters can create slow change stimuli out of a wide variety of pictures, and it allows experimenters to use these stimuli in systematic studies where participants complete many slow change trials in a row (although we did not use that option here). As such, this approach provides researchers with the tools to address a range of questions surrounding change blindness. To illustrate the potential of this approach, we will briefly touch on one obvious question: What are the conditions that render observers blind to slow changes? Given the evidence for a role of attention in change detection, perception, and memory (Henderson, Hayes, Peacock, & Rehrig, 2019; Hollingworth & Henderson, 2000) and for a role of image semantics in attention allocation (Shomstein, Malcolm, & Nah, 2019), in an exploratory analysis we examined whether the internal representations of different slow change videos differed and whether this can be related to their semantic properties. In particular, in some videos, the large area undergoing a color change was a nameable object central to the gist of the scene (boat, car), whereas in others the color change area was more accurately described as part of the background (wall, floor) and therefore less semantically relevant. We were interested in whether the pattern of “same” responses across cue and color conditions would differ depending on the semantic relevance of the changing item or the semantic relevance of the color of the item. To informally summarize our findings, we found substantial differences in these patterns between videos (such as increased proportions of “same” responses to “initial” for some videos, a decreased proportion of “same” responses to “initial” in other videos, and even tentative evidence of guessing on some videos) but grouping videos into an object category and a background category (based on the authors’ subjective judgment) did not explain the pattern of differences. Nevertheless, this exploratory analysis does illustrate the potential of this recently proposed approach to change blindness research. Although this particular study was not designed with an analysis of stimulus content in mind, our approach renders it straightforward to create a larger set of change blindness stimuli that systematically differ in semantic (or other) properties and to more fruitfully apply analyses such as this one.

In general, the present work offers an understanding of the memory representations formed during a slowly changing image—a first step in determining how slow change blindness arises—and illustrates how useful studying slow change blindness can be for learning about change blindness in general, as well as for scene perception, memory, attention, and more.
